# Revision of the Oriental genus *Horniella* Raffray (Coleoptera, Staphylinidae, Pselaphinae) – Supplementum 1

**DOI:** 10.3897/zookeys.506.9204

**Published:** 2015-06-01

**Authors:** Zi-Wei Yin, Li-Zhen Li

**Affiliations:** 1 Department of Biology, College of Life and Environmental Sciences, Shanghai Normal University, 100 Guilin Road, Shanghai, 200234, P. R. China

**Keywords:** Pselaphinae, *Horniella*, new species, new record, China, Myanmar

## Abstract

Two new species of the genus *Horniella* Raffray are described from China: *Horniella aculeata* **sp. n.** (Yunnan Province) and *Horniella jinggangshana* **sp. n.** (Jiangxi Province). *Horniella nakhi* Yin & Li is recorded from a new locality. Three potentially new species from Myanmar and China, each represented by female specimen(s) only, are left unnamed. Their collecting data are given, and the genital complex figured for future comparison.

## Introduction

Since the publication of our revision of the genus *Horniella* Raffray (Yin and Li 2014), we have had the opportunity to examine additional material collected in China and Myanmar. A study of this material revealed two new species, a new locality for *Horniella nakhi* Yin & Li, and the first record of the genus from Myanmar. The material also includes three possibly different species represented only by females that are kept unnamed until associated males become available in the future.

## Material and methods

The methods, terminology, and abbreviations applied are the same as in Yin and Li 2014. Authors’ supplementary notes are included in brackets.

Material treated in this study is housed in the following public institution and museums:

MSNG Museo Civico di Storia Naturale “Giacomo Doria”, Genova, Italy (Roberto Poggi);

NSMT National Museum of Nature and Science, Tokyo, Japan (Shûhei Nomura);

SNUC Insect Collection of the Shanghai Normal University, Shanghai, China (Zi-Wei Yin).

## Species treatment

### Horniella aculeata

Taxon classificationAnimaliaColeopteraStaphylinidae

Yin & Li sp. n.

http://zoobank.org/2E67E322-EC41-4D79-8166-CAD99F352B77

Figs 1A , 2 , 5A

#### Type material

(2 ♂♂, 4 ♀♀)**. Holotype: China**: ♂, labeled ‘Mengla Ziranbaohuqu {勐腊自然保护区}, (Xishuangbanna) {西双版纳}, S. Yunnan, China, Sept. 13th, 1993, Coll. Y. Watanabe / HOLOTYPE {red} ♂, *Horniella aculeata* sp. n., det. Yin & Li, 2015, NSMT’ (NSMT). **Paratypes: China**: 3 ♀♀, same label data as holotype (NSMT); 1 ♂, 1 ♀, labeled ‘Tropical Rainforest (Tropical Botanical Garden) {热带植物园}, Menglun {孟仑}, Mengla County {勐腊县} / (Xishuangbanna), S. Yunnan, China, Oct. 29th, 1992, Coll. Y. Watanabe.’ (SNUC). Each paratype bears a type label as: ‘PARATYPE {yellow} ♀ {or ♂}, *Horniella aculeata* sp. n., det. Yin & Li, 2015, NSMT {or SNUC}’.

#### Description.

**Figure 1.**
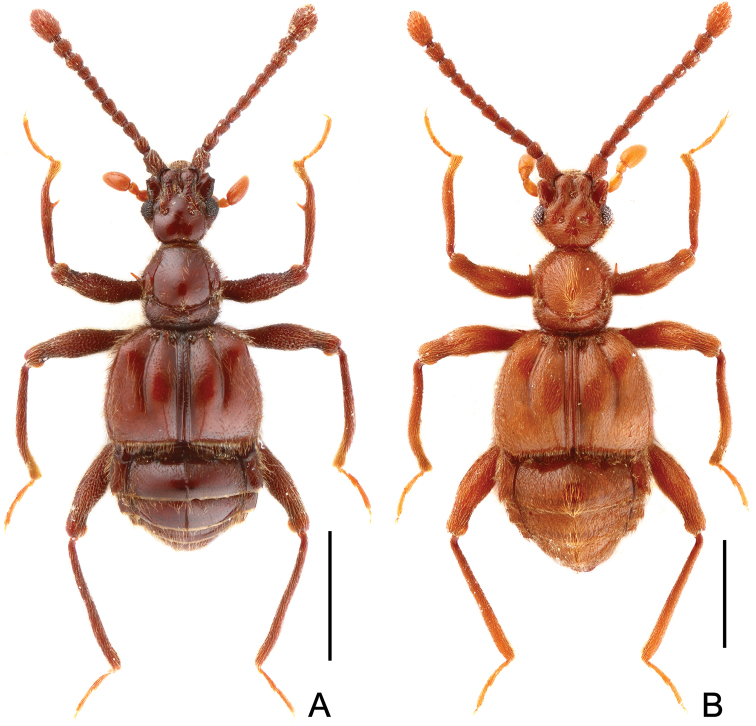
Male habitus of *Horniella* species. **A** *Horniella aculeata* **B** *Horniella jinggangshana*. Scales: 1.0 mm.

**Figure 2.**
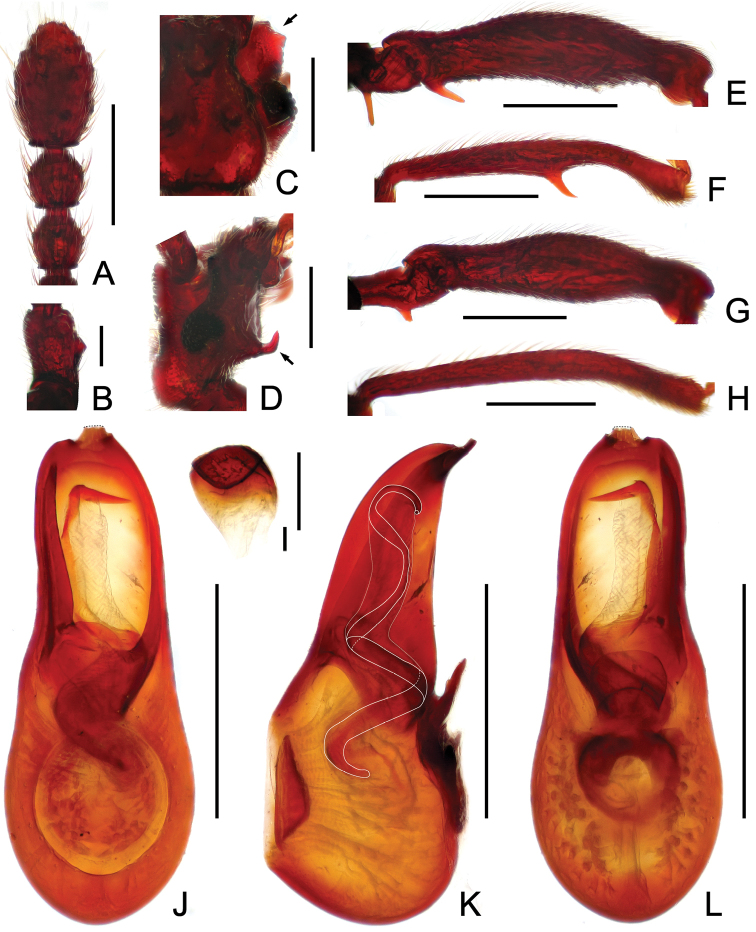
Male diagnostic features of *Horniella aculeata* **A** antennal club **B** scape **C** right half of the head, in dorsal view **D** head, in lateral view **E** protrochanter and profemur **F** protibia **G** mesotrochanter and mesofemur **H** mesotibia **I** sternite IX **J** aedeagus, in dorsal view **K** same, in lateral view **L** same, in ventral view. Scales: all = 0.3 mm, except **B, I** = 0.1 mm.

Male (Fig. 1A). Length 2.95–3.0 mm. Head slightly wider than long, HL 0.54–0.58 mm, HW 0.61–0.62 mm; anterolateral genal projections (Fig. 2C) distinct, anterior margins evenly concave; median sulcus between antennal tubercles short and deep; scapes (Fig. 2B) acutely expanded at basolateral margins; clubs (Fig. 2A) loosely formed by apical three moderately enlarged antennomeres; head venter with pair of short, strongly curved lateral spines (Fig. 2D). Maxillary palpomeres II stout, broadened at middle. Each eye composed of about 30 facets. Pronotum slightly longer than wide, PL 0.63–0.64 mm, PW 0. 59–0. 61 mm. Elytra wider than long, EL 0.82–0.85 mm, EW 1.23–1.25 mm; discal striae reaching apical 2/3 of elytral length. Protrochanters and profemora (Fig. 2E) each with one distinct ventral spine, protibiae (Fig. 2F) with mesal margins strongly arcuate at apical half, with large sharp spine at mesal margin near middle; mesotrochanters (Fig. 2G) each with one short, blunt ventral protuberance, mesofemora simple, mesotibiae (Fig. 2H) simple; tarsomeres II normal, not extending to beneath tarsomeres III. Abdomen large, AL 0.94–0.95 mm, AW 1.19–1.26 mm; tergite IV (first visible tergite) with median carina extending to half tergal length or slightly more, lateral discal carinae short; tergite V lacking median carina. Sternite IX (Fig. 2I) nearly oval, with well-sclerotized apical half and membranous basal half. AeL 0.62 mm; aedeagus (Fig. 2J–L) with slightly asymmetric median lobe slightly curved rightwards in dorso-ventral view; endophallus composed of one conspicuously long, partly membranous, and twisted sclerite with pointed, curved apex.

Female. Similar to male in general appearance; scapes not expanded at basolateral margins; each eye composed of about 25 facets; profemora each with two subequal ventral spines near base, protibiae lacking spine, not strongly arcuate at apical half; mesotrochanters lacking ventral spine. BL 2.81–2.94 mm, HL 0.65–0.69 mm, HW 0.57–0.58 mm, PL 0.62–0.63 mm, PW 0.59–0.61 mm, EL 0.70–0.74 mm, EW 1.12–1.16, AL 0.84–0.88 mm, AW 1.22–1.23 mm. Genital complex (Fig. 5A) 0.36 mm wide, with slightly sclerotized, transverse apical portion, and with membranous, elongate basal portion.

#### Differential diagnosis.

The new species is placed as a member of the *Horniella burckhardti* group (for diagnoses of species-groups refer to Yin and Li 2014). Males of *Horniella aculeata* have the aedeagal endophallus composed of one elongate sclerite, similar to that of *Horniella hongkongensis* Yin & Li. The two species can be readily separated by 1) the more distinct and acute protuberance on the mesal margins of the scapes, 2) the oblique ventral spine at base of the profemora, and 3) the presence of a large, sharp spine on the mesal margins of the protibiae in *Horniella aculeata*. In *Horniella*, the presence of a spine on the mesal margin of the protibiae only occurs in *Horniella simplaria* Yin & Li which belongs to the *Horniella hirtella* group, otherwise the two species are easily separable from each other.

#### Distribution.

Southwestern China: Yunnan.

#### Etymology.

The specific epithet refers to the acute spine on the protibia.

### Horniella jinggangshana

Taxon classificationAnimaliaColeopteraStaphylinidae

Yin & Li sp. n.

http://zoobank.org/F79959DA-392F-42A1-86BA-BF8F0C1172BC

Figs 1B , 3 , 5B–C

#### Type material

(1 ♂, 2 ♀♀)**. Holotype: China**: ♂, labeled ‘China: W. Jiangxi, Ji’an City, Jinggang Shan N. R. {井冈山自然保护区}, Shuikou {水口}, 26°32'42"N, 114°06'03"E, mixed leaf litter, sifted, 790–900 m, 30.vii.2014, J.Y. Hu / HOLOTYPE {red} ♂, *Horniella jinggangshana* sp. n., det. Yin & Li, 2015, SNUC’ (SNUC). **Paratypes: China**: 2 ♀♀, same label data as holotype (SNUC). Each paratype bears a following label: ‘PARATYPE {yellow} ♀, *Horniella jinggangshana* sp. n., det. Yin & Li, 2015, SNUC’.

#### Description.

**Figure 3.**
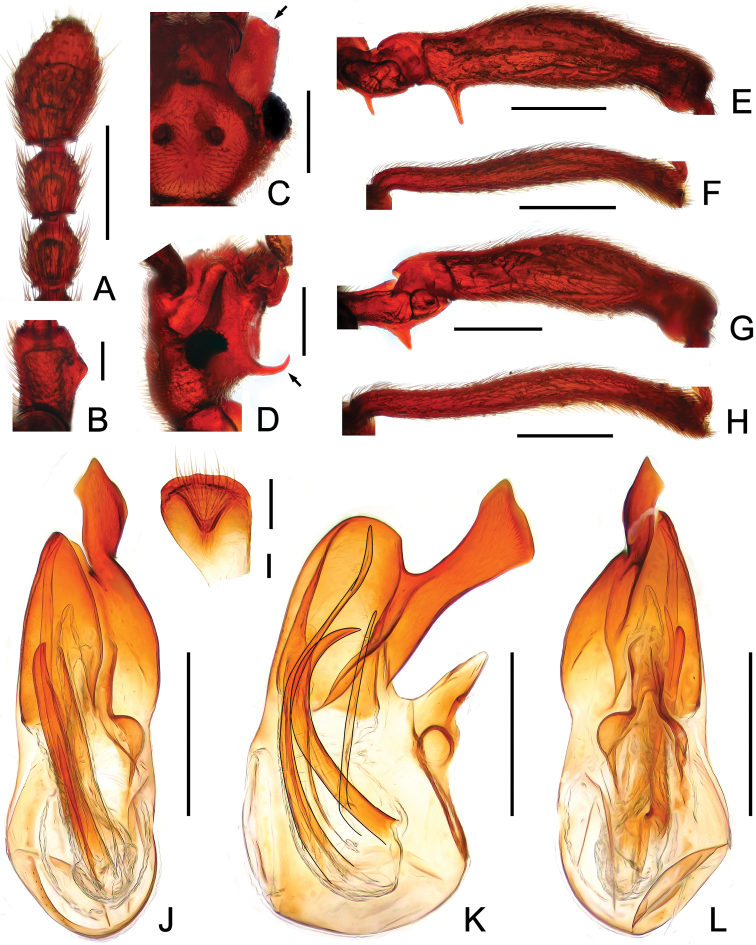
Male diagnostic features of *Horniella jinggangshana* **A** antennal club **B** scape **C** right half of the head, in dorsal view **D** head, in lateral view **E** protrochanter and profemur **F** protibia **G** mesotrochanter and mesofemur **H** mesotibia **I** sternite IX **J** aedeagus, in dorsal view **K** same, in lateral view **L** same, in ventral view. Scales: all = 0.3 mm, except **B**, **I** = 0.1 mm.

Male (Fig. 1B). Length 3.67 mm. Head as long wide, HL 0.75 mm, HW 0.75 mm; anterolateral genal projections (Fig. 3C) distinct, anterior margins slightly concave; median sulcus between antennal tubercles short and moderately deep; scapes (Fig. 3B) angularly expanded at basolateral margins; clubs (Fig. 3A) loosely formed by apical three moderately enlarged antennomeres; venter with pair of markedly long, curved lateral spines (Fig. 3D). Maxillary palpomeres II stout, broadened at middle. Each eye composed of about 35 facets. Pronotum slightly longer than wide, PL 0.76 mm, PW 0.72 mm. Elytra wider than long, EL 0.94 mm, EW 1.46 mm; discal striae reaching more than apical 2/3 of elytral length. Protrochanters and profemora (Fig. 3E) each with one distinct ventral spine, protibiae (Fig. 3F) each with short apical protuberance; mesotrochanters (Fig. 3G) each with one big ventral spine, mesofemora simple, mesotibiae (Fig. 3H) with small preapical denticles and short apical projection; tarsomeres II normal, not extending to beneath tarsomeres III. Abdomen large, AL 1.22 mm, AW 1.41 mm; tergite IV (first visible tergite) with short median carina, lacking lateral discal carinae; tergite V lacking median carina. Sternite IX (Fig. 3I) nearly oval, with well-sclerotized apical half and membranous basal half. AeL 0.82 mm; aedeagus (Fig. 3J–L) with left half of median lobe greatly protruding in dorso-ventral view; endophallus composed of three long, curved sclerites.

Female. Similar to male in general appearance; scapes not expanded at basolateral margins; each eye composed of about 35 facets; profemora each with two subequal ventral spines near base, protibiae lacking preapical protuberance; mesotrochanters lacking ventral spine, mesotibiae lacking apical projection; tergite VII with posterior margin protruding at middle. BL 3.53–3.58 mm, HL 0.65–0.69 mm, HW 0.66–0.72 mm, PL 0.66–0.70 mm, PW 0.65–0.66 mm, EL 0.92–0.93 mm, EW 1.30–1.37, AL 1.26–1.30 mm, AW 1.33–1.34 mm. Genital complex (Fig. 5B–C) 0.43 mm wide, with broad apical portion, and coil-shaped basal portion.

#### Differential diagnosis.

The new species is placed as a member of the *Horniella centralis* group. Its closest congener is probably *Horniella falcis* Yin & Li, which is known from a single female. The females of these two species share a similar habitus, the protruding posterior margin of tergite VII, and a similar form of the genital complex. They can be tentatively separated by the less protruding posterior margin of tergite VII, the broader genital complex in dorsal-ventral view in *Horniella jinggangshana*, and their distributions (straight line distance of separation measures ca. 710 km). The males of *Horniella jinggangshana* can be readily separated from all other congeners by the unique form of the aedeagus and structure of the endophallus.

#### Distribution.

Eastern China: Jiangxi.

#### Etymology.

The new species is named after its type locality, the Jinggang Shan Nature Reserve.

### Horniella nakhi

Taxon classificationAnimaliaColeopteraStaphylinidae

Yin & Li

Fig. 5D–E

Horniella nakhi Yin & Li, 2014: 25.

#### Material examined.

1 ♂, 1 ♀, labeled ‘Mt. Jizu Shan {鸡足山, ca. 25°58'N, 100°23'E } (2130 m), Binchuan {宾川县}, NW Yunnan, China, 25.X.1995, Coll. Y. Watanabe & Xiao N. / *Horniella nakhi* Yin & Li, 2014, det. Z.W. Yin, 2015’ (NSMT).

#### Distribution.

This species was known from one male and two females collected in Naxi Autonomous County. The present record extends its distribution to the Jizu Mountain, ca. 90 km south from the type locality.

#### Comments.

The population from Jizu Mountain exhibits a stouter aedeagal form and different structure of the endophallus (Fig. 5D–E). These are attributed to intraspecific variation because all other male diagnostic features, e.g. the strongly projecting apical portion of the protibiae, seem quite stable.

### Horniella sp. 1

Taxon classificationAnimaliaColeopteraStaphylinidae

Figs 4A , 5F, G

#### Material examined.

3 ♀♀, labeled ‘Carin, Asciuii Chebà, 1200–1300 m, L. Fea. III-IV.{18}88. / Museo, Civico, di Genova; 1 ♀, same data, except for ‘I - 88’ (MSNG). Each specimen bears a following label: ‘cf. *Horniella* sup. 1., *Horniella* sp. 1, det. Z.W. Yin, 2015’.

#### Measurements.

**Figure 4.**
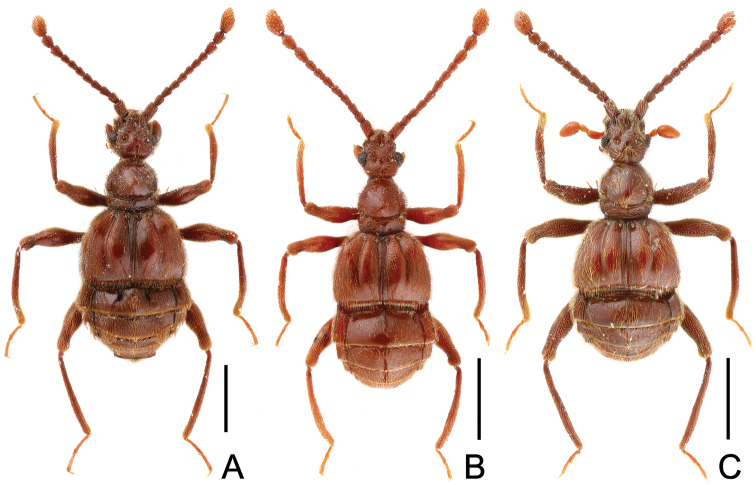
Male habitus of *Horniella* species. **A** *Horniella* sp. 1 **B** *Horniella* sp. 2 **C** *Horniella* sp. 3. Scales: 1.0 mm.

Female (Fig. 4A). BL 3.70–3.78 mm, HL 0.80–0.81 mm, HW 0.72–0.73 mm, PL 0.76–0.78 mm, PW 0.74–0.75 mm, EL 1.0–1.02 mm, EW 1.48–1.52 mm, AL 1.11–1.20 mm, AW 1.59–1.60 mm. Each eye composed of about 38 facets. Width of genital complex 0.37 mm.

#### Distribution.

Eastern Myanmar: Kayah State.

#### Comments.

**Figure 5.**
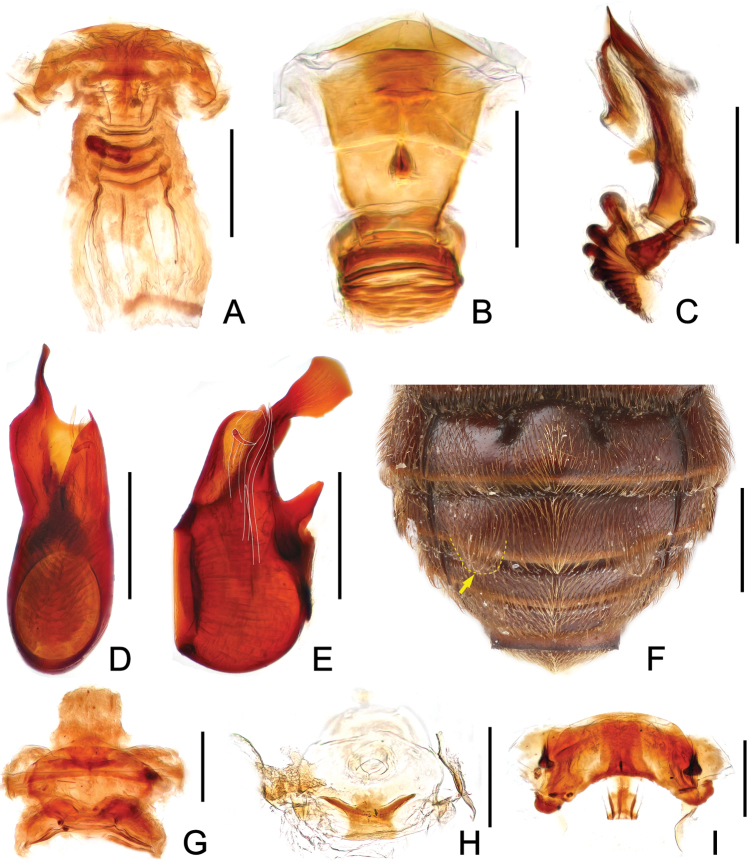
Details of *Horniella* species (**A** *Horniella aculeata* **B–C** *Horniella jinggangshana* **D–E** *Horniella nakhi* **F–G** *Horniella* sp. 1 **H** *Horniella* sp. 2 **I** *Horniella* sp. 3). **A, B, G, H, I** female genital complex, in dorsal view **C** same, in lateral view **D** aedeagus, in dorsal view **E** same, in lateral view **F** abdomen, in dorsal view. Scales: all = 0.2 mm, except **F** = 0.5 mm.

The large body size combined with the unique setation on tergite V (Fig. 5F) clearly indicates a new species. The female genital complex (Fig. 5G) is here illustrated for reference to future study.

### Horniella sp. 2

Taxon classificationAnimaliaColeopteraStaphylinidae

Figs 4B , 5H

#### Material examined.

2 ♀♀, labeled ‘Carin, Asciuii Chebà, 900–1100 m, L. Fea. V XII-{18}88. / Museo, Civico, di Genova / cf. *Horniella* sup. 1., *Horniella* sp. 2, det. Z.W. Yin, 2015’ (MSNG).

#### Measurements.

Female (Fig. 4B). BL 3.09 mm, HL 0.61–0.64 mm, HW 0.57–0.58 mm, PL 0.66–0.67 mm, PW 0.57–0.58 mm, EL 0.79–0.80 mm, EW 1.19–1.20 mm, AL 1.00–1.01 mm, AW 1.23–1.25 mm. Each eye composed of about 40 facets. Width of genital complex 0.33 mm.

#### Distribution.

Eastern Myanmar: Kayah State.

#### Comments.

This material represents the first record of the *Horniella hirtella* group in Myanmar. The form of the genital complex (Fig. 5H) is highly similar to that of *Horniella philippina* Yin & Li (Yin and Li 2014: fig. 49F), but the Myanmar population probably represents a different species insomuch as its distribution is considered, as well as the presence of a much longer median carina on tergite IV.

### Horniella sp. 3

Taxon classificationAnimaliaColeopteraStaphylinidae

Figs 4C , 5I

#### Material examined.

1 ♀, labeled ‘Guibeishan (450 m), (Tull.), Yaoshan Xiang, Libo Xian / [Guizhou, CHINA], 中国贵州省荔波县瑶山乡, 11.ix.1997, T. Kishimoto / cf. *Horniella* sup. 1., *Horniella* sp. 3, det. Z.W. Yin, 2015’ (NSMT).

#### Measurements.

Female (Fig. 4C). BL 3.15 mm, HL 0.74 mm, HW 0.60 mm, PL 0.66 mm, PW 0.64 mm, EL 0.81 mm, EW 1.31 mm, AL 0.94 mm, AW 1.32 mm. Each eye composed of about 22 facets. Width of genital complex 0.46 mm.

#### Distribution.

Southwestern China: Guizhou.

#### Comments.

The unique form of the genital complex and distribution of this female indicate a different species. Illustration of its genital complex (Fig. 5I) is provided for future comparison.

## Supplementary Material

XML Treatment for Horniella aculeata

XML Treatment for Horniella jinggangshana

XML Treatment for Horniella nakhi

XML Treatment for Horniella sp. 1

XML Treatment for Horniella sp. 2

XML Treatment for Horniella sp. 3
